# Infection-Related Death among Persons with Refractory Juvenile Idiopathic Arthritis

**DOI:** 10.3201/eid2210.151245

**Published:** 2016-10

**Authors:** Mario Abinun, Jonathan P. Lane, Mark Wood, Mark Friswell, Terence J. Flood, Helen E. Foster

**Affiliations:** Great North Children's Hospital, Newcastle upon Tyne, UK (M. Abinun, J.P. Lane, M. Friswell, T.J. Flood, H.E. Foster);; Newcastle University, Newcastle upon Tyne (M. Abinun, H.E. Foster);; Leeds General Infirmary, Leeds, UK (M. Wood)

**Keywords:** Juvenile idiopathic arthritis, combined immunosuppressive and antiinflammatory therapy, biological disease-modifying drugs, anti–TNF-α, TNF-α blocking agents, central venous catheter bacteria, children, United Kingdom, bacteria, sepsis, immunosuppression

## Abstract

Severe infections are emerging as major risk factors for death among children with juvenile idiopathic arthritis (JIA). In particular, children with refractory JIA treated with long-term, multiple, and often combined immunosuppressive and antiinflammatory agents, including the new biological disease-modifying antirheumatic drugs (DMARDs), are at increased risk for severe infections and death. We investigated 4 persons with JIA who died during 1994–2013, three of overwhelming central venous catheter–related bacterial sepsis caused by coagulase-negative *Staphylococus* or α-hemolytic *Streptococcus* infection and 1 of disseminated adenovirus and Epstein-Barr virus infection). All 4 had active JIA refractory to long-term therapy with multiple and combined conventional and biological DMARDs. Two died while receiving high-dose systemic corticosteroids, methotrexate, and after recent exposure to anti–tumor necrosis factor-α biological DMARDs, and 2 during hematopoietic stem cell transplantation procedure. Reporting all cases of severe infections and especially deaths in these children is of paramount importance for accurate surveillance.

## Abstract 2

Bacterial sepsis led to multiorgan failure in persons receiving immunosuppressive and antiinflammatory drugs.

Juvenile idiopathic arthritis (JIA), a group of clinically heterogonous conditions with arthritis of unknown origin beginning before 16 years of age and persisting for >6 weeks, is the most common childhood chronic rheumatic disorder (*1*). The most severe forms are those with systemic (So-JIA) or polyarticular (poly-JIA) onset, progressing to polyarticular disease. Despite the success of conventional and new biological disease-modifying antirheumatic drugs (DMARDs), a substantial percentage (>30%) of patients will have ongoing active disease into adulthood that includes sequelae from chronic inflammation and considerable morbidity from joint damage, osteoporosis, growth retardation, psychosocial morbidity, and reduced quality of life and education or employment (*2*).

Rapidly evolving guidelines (*3*) include the window-of-opportunity concept, where biological DMARDs are used as tailored therapy, depending on the disease category, and in naive patients not previously treated with conventional DMARDs (e.g., corticosteroids) (*2*). In the United Kingdom, treatment guidelines are regulated by the National Institute for Clinical Excellence, which specifies indications for tumor necrosis factor-α (TNF-α), interleukin (IL) 6, T-cell activation, and IL-1 blocking agents (https://www.nice.org.uk/guidance/ta373; https://www.nice.org.uk/advice/esnm36). However, no good evidence exists to guide clinicians when they confront failure of the initial biological DMARD (*4*), and switching to a second (or third) (*5*) or combining >2 biological DMARDs (*6*) raises concern about risks from severe infections and development of malignancy and new autoimmune disorders (*7*,*8*). For this small group of patients with refractory JIA, hematopoietic stem cell transplantation (HSCT) might be the only treatment option (*9*).

We report details of 4 patients referred for HSCT because of refractory JIA who died of overwhelming infection from central venous catheter (CVC)–related bacterial sepsis (3 patients) or disseminated viral infection (1 patient). Two died while receiving high-dose systemic corticosteroids and methotrexate and after recent exposure to anti–TNF-α agents, before they underwent stem cell collection, and were not started on conditioning chemotherapy at the time of death; 2 died during HSCT (*10*,*11*).

## Case Reports

Parents provided informed consent for data and tissue collection. Consent was obtained when the child started treatment with new anti–TNF-α agents (British Society for Paediatric and Adolescent Rheumatology database) or at assessment for HSCT (Newcastle upon Tyne Hospitals National Health Service [NHS] Foundation Trust, Newcastle upon Tyne, UK).

### Patient 1

Patient 1, a 13-year-old girl with refractory So-JIA who was previously reported with osteoarticular tuberculosis while treated with etanercept (*12*) (Table 1), never achieved complete disease control (Juvenile Arthritis Disease Activity Score [JADAS]–10 score 25–30) (Table 1) (*13*). She was assessed for HSCT on February 17, 2004 (Table 2). After CVC insertion for treatment with weekly intravenous methylprednisolone pulses (IVMPs) and 1 dose of infliximab, routine CVC cultures grew fully sensitive coagulase-negative *Staphylococcus*. She was treated with systemic teicoplanin and vancomycin locks to all 3 CVC lumens for 7 days (*15*). On February 28, three days after antimicrobial treatment ended, she was readmitted with fever (40°C), generalized macular erythematous rash, abdominal discomfort, nausea, vomiting, and diarrhea. Alongside empirical treatment with systemic antimicrobial drugs (vancomicin and cefotaxime) and intravenous fluids, presumed adrenal insufficiency was treated with hydrocortisone, but she remained febrile (38.6°C); on February 29, she had a sudden episode of hypotension (blood pressure 70 mm Hg) requiring fluid bolus resuscitation and further hydrocortisone. On March 1, as the CVC was accessed for administration of antimicrobial drugs, she became pale and light-headed and collapsed; the CVC was removed, but she died of cardiorespiratory arrest. Multiple peripheral blood and CVC cultures taken during this period remained sterile. Autopsy showed congested lungs and pleural effusions, atrophic adrenal glands, no identifiable thymus, and no erythrophagocytosis in liver or spleen. Cultures from the CVC tip, both lungs, and the pleural fluid samples taken post mortem grew α-hemolytic *Streptococcus*.

**Table 1:** Disease characteristics and treatment at time of death for 2 children with JIA, Newcastle upon Tyne, United Kingdom*

Characteristic	Patient 1	Patient 2
Year of disease onset/year of death	1996/2004	2000/2004
Age at So-JIA diagnosis/at death, y	5/13	2/6
JIA symptom or sign, ever		
Fever	Yes	Yes
Rash	Yes	Yes
Arthritis	Yes	Yes
Lymphadenopathy	No	No
Hepato/splenomegaly	Yes	Yes
Serositis	No	No
Macrophage activation syndrome	No	Yes
Disease remission, ever	No	No
JADAS-10†	20–35	12–32
Treatment		
Corticosteroids‡	Yes§	Yes§
Methotrexate¶	Yes§	Yes§
Cyclosporin A	No	Yes#
Intravenous immunoglobulins**	No	Yes††
Etanercept	Yes‡‡	No
Infliximab	Yes§,§§	Yes§¶¶
Side effects of treatment		
Cushingoid	Yes	Yes
Cataracts	Yes	No
Osteoporosis	Yes	Yes
Osteoarticular tuberculosis	Yes	No
Stunted growth	Yes	Yes

*JADAS, Juvenile Arthritis Disease Activity Score; JIA, juvenile idiopathic arthritis; So-JIA, systemic JIA. †Linear sum of the scores of the 4 JADAS components (0–40): physician global assessment of disease activity (measured on a 10-cm visual analog scale; 0 = no activity and 10 = maximum activity); parent/patient global assessment of well-being (measured on a 10-cm visual analog scale; 0 = very well and 10 = very poor); count of joints with active disease (0–10); erythrocyte sedimentation rate (actual value/10; range 0–10) (*13*). ‡Methylprednisolone pulses (intravenous 30 mg/kg/day; maximum. 1 g) given as a 3-day course or a single dose (weekly or otherwise), when indicated based on clinical decision (for treating a disease flare). Prednisolone maintenance dose (orally 0.5–1 mg/kg/d) with adjusting and aiming for alternate day regimen whenever possible depending on the clinical course or disease activity. §Treatment at time of death. ¶Subcutaneously 15 mg/m^2^/wk. #March–December 2002. **2 g/kg/mo. ††December 2003–January 2004. ‡‡0.4 mg/kg/wk during March–May 2000 (*12*) and again during May 2003–January 2004. §§5 mg/kg single dose, February 2004. ¶¶6 mg/kg/mo during December 2002–December 2003.

**Table 2:** Laboratory parameters of inflammatory activity for 2 children with juvenile idiopathic arthritis, Newcastle upon Tyne, United Kingdom, 2004*

Patient no., date	CRP, mg/dL	Ne, × 10^9^ cells/L	PLT, × 10^9^/L	ESR, mm/h	Ferr, μg/L	Fib, g/L	TG, mmol/L	Alb, g/L	ALT, U/L	Na, mmol/L
Patient 1										
Feb 17	100	20.2	322	35	485	5.4	0.7	34	24	142
Feb 28	107	28.5	266	–	–	–	–	31	35	136
Feb 29	157	15.8	140	–	–	–	–	22	48	134
Mar 1	63	7.1	79	–	–	–	–	23	120	140
Patient 2										
May 27	122	17.2	475	45	339	6.3	1.9	44	10	135
Jun 1	260	34.9	603	–	2815	8.0	–	38	23	137
Jun 6	365	37.5	710	105	7692	8.1	–	37	50	134
Jun 10	127	70.5	244	–	–	2.9	–	13	125	134

*Laboratory parameters for diagnosing macrophage activation syndrome: Ferr >684 μg/L and any 2 of the following signs: PLT count <181 × 10^9^/L, ALT >48 U/L, TG >156 mg/dL, fibrinogen <360 mg/dL (*14*). See section in text on MAS versus Multiorgan Failure. Alb, serum albumin level (reference 38–53 g/L); ALT, serum alanine transaminase level (reference 0–40 U/L); CRP, C-reactive protein (reference 0–5 mg/dL); ESR, erythrocyte sedimentation rate (Westergren; reference 1–10 mm/h); Ferr, serum ferritin level (reference 20–60 μg/L); Fib, serum fibrinogen level (reference 1.5–4.0 g/L); Na, serum sodium level (reference 135–145 mmol/L); Ne, absolute neutrophil count; PLT, absolute platelet count; TG, serum triglyceride level (reference 0.5–1.8).

### Patient 2

Patient 2, a 6-year-old girl, had refractory So-JIA (Table 1) with macrophage activation syndrome (MAS), a form of hemophagocytic lymphohistiocytosis and a potentially fatal complication characterized by unremitting fever, pancytopenia, liver failure with coagulopathy, and central nervous system dysfunction (*14*). Several months before referral, she was brought for care again with possible MAS; symptoms and signs included fever, hepatomegaly, diarrhea, anemia, thrombocytopenia, high serum ferritin (5,670 μg/L [reference 20–60 μg/L]), albeit with leukocytosis and normal clotting (*16*). She was treated with IVMP and high-dose intravenous immunoglobulin. A previously inserted CVC was removed because of *Enterobacter intermedius* infection. Because her disease was never in full remission (JADAS-10 score 20–30; Table 1), she was referred on May 27, 2004, for HSCT (Table 2). On June 1 (one week after new CVC insertion), she was admitted with fever (38°C), macular erythematous rash, vomiting, swelling and pain of several joints, and cough (Table 2). Chest examination and radiographic findings were normal, and she was treated empirically with systemic antimicrobial drugs (teicoplanin and meropenem) for 1 week (*15*) and a 3-day course of IVMP for presumed MAS. After transient improvement during the next few days, on June 6 the child again became unwell, with fever (38.5°C), rash, hepatomegaly, and joint pain (Table 2). Empirical treatment with systemic antimicrobial drugs (teicoplanin and ceftriaxone) was restarted with another 3-day course of IVMP, but she remained febrile (38.5°C) and became restless with abdominal pain and vomiting. She was transferred to the pediatric intensive care unit (PICU), where surgical reasons for acute abdomen pain were excluded. Ciprofloxacin and ambisome were added to the treatment regimen, but the child’s condition rapidly progressed into multiorgan failure (Table 2), and she died on June 10. Multiple peripheral blood and CVC cultures taken during this period remained sterile. The family did not agree to autopsy. Culture from the CVC tip removed postmortem grew coagulase-negative *Staphylococcus*.

### Patients 3 and 4

Both children received immunosuppressive conditioning with anti–T-cell globulin (rabbit, 10 mg/kg), fludarabine (150 mg/m^2^), and cyclophosphamide (120 mg/kg) (*10*,*11*). Both died during T-cell–depleted (by CD34+ positive selection; Miltenyi Biotec, San Diego, CA, USA) autologous HSCT.

#### Patient 3

Patient 3 was an 18-year-old woman whose So-JIA was diagnosed at 10 years of age. She had active systemic disease, MAS, and progressive polyarthritis refractory to conventional (corticosteroids, methotrexate, cyclosporine, leflunomide) and biological DMARDs, both TNF-α (infliximab, etanercept) and IL-1 (anakinra) inhibitors (JADAS-10 score over the years 22–35). She underwent HSCT in 2007 with MAS prevention (prednisolone, cyclosporine, and anakinra) during conditioning, but after uneventful engraftment, she died 2.5 months after HSCT of disseminated adenovirus (blood, feces, brain) and Epstein-Barr virus (EBV) (blood, cerebrospinal fluid) infection/reactivation. *Aspergillus fumigatus* was grown from a paranasal sinus washout sample in terminal stage; autopsy was not performed (*10*).

#### Patient 4

Patient 4 was a 13-year-old girl whose rheumatoid factor + poly-JIA was diagnosed at 4 years of age. She had progressive and debilitating polyarthritis refractory to conventional (corticosteroids, methotrexate) and biological (infliximab, etanercept, anakinra, rituximab) DMRADSs (JADAS-10 score over the years 24–30). She underwent HSCT in 2009. α-hemolytic *Streptococcus* grew from CVC culture taken during a febrile episode after receipt of anti–T-cell globulin, and she was treated empirically with meropenem and teicoplanin; unusually, she rapidly progressed into multiorgan failure requiring ventilatory, inotropic, and renal support in the PICU. Because results of initial liver function tests, including clotting, were normal, and C-reactive protein (CRP) response was adequate, the impression was of bacterial (or fungal) septicemia and renal failure. After transient improvement, she completed conditioning and HSCT and, despite renal failure, maintained stable neutrophil engraftment but remained platelet dependent. Bone marrow biopsy was hypocellular and showed some evidence of macrophage activation. Subsequently, and in parallel with acute pancreatitis, encephalopathy, and progressive enteral and liver failure, the girl manifested prolonged hyperinflammatory response (CRP 100–170 mg/L [reference 0–5 mg/L]; fibrinogen 6–10 g/L [reference 1.5–4.0 g/L]; raised neutrophil count >20 × 10^9^ cells/L) despite broad-spectrum antimicrobial and antifungal therapy. Multiple cultures and viral PCRs from different sites (blood, CVC, and other line tips; bone marrow and intestine biopsy; cerebrospinal fluid; maxillary sinus washing) remained negative. She died on day 43 after HSCT; autopsy confirmed multiorgan failure with severe secondary pancreatitis (*11*).

## Possible Risk Factors for Severe Infection and Death

### Treatment with Combined DMARDs, Including Biologicals

At death, patients 1 and 2 had active disease treated with high-dose systemic corticosteroids and methotrexate; they previously had been exposed to long-term and multiple DMARDs, including anti–TNF-α biologicals (Table 1). Patients 3 and 4 underwent autologous T-cell–depleted HSCT after a severely immunosuppressive conditioning regimen.

Clinical observation of increased risk from severe infections in children with JIA, often requiring treatment in a hospital (*17*), recently was confirmed in a study of a large JIA cohort (*18*). The increased risk for concurrent immunosuppressive therapy, in addition to the underlying disease-related immune dysfunction (*8*,*17*), was supported by this study, in which high-dose systemic corticosteroids, but not methotrexate and/or anti–TNF-α agents, substantially increased susceptibility to severe infections (*18*). Although simultaneous use of different biological DMARDs is not common (5,6), a recently published study highlighted the exposure to multiple and often combined immunosuppressive drugs, such as corticosteroids, methotrexate, cyclosporine, cyclophosphamide, and a variety of biological antiinflammatory DMARDs, including TNF-α, IL-1, and IL-6 blocking agents, as a major risk factor for severe infections and the unusually high rate of death for a selected group of children with refractory So-JIA, often complicated by MAS, and associated with pulmonary hypertension, interstitial lung disease, and alveolar proteinosis (*19*). It is well recognized that severe immunosuppression targeting B- and/or T-lymphocyte functions (e.g., B-cell–depleting agents, such as rituximab, T-cell activation blocking agent abatacept, anti-CD52 monoclonal antibody alemtuzumab, HSCT procedure) is often complicated with infections caused by a wide spectrum of pathogens, such as pyogenic bacteria, viruses, and fungi (*7*,*9*–*11*). In contrast, blocking specific inflammatory pathways (e.g., IL-1, IL-6, TNF-α) mimics some of the very rare primary immunodeficiencies of the innate immune system (*20*), with susceptibility to a relatively narrow range of pathogens (*21*,*22*). Infections with encapsulated pyogenic bacteria have been reported in patients with deficiencies of IL-6 function (*23*), whereas the essential role of TNF-α in defense against intracellular pathogens (*24*) was highlighted by the initially observed increased risk for mycobacterial infections associated with anti–TNF-α biologicals (*7*,*12*), prompting the introduction of effective screening and prevention measures (*24*). Although inconsistently reported (*25*), unusually prolonged, severe, and life-threatening infections with common bacterial pathogens are being seen in children with JIA treated with combined DMARDs, including anti–TNF-α agents (*26*,*27*). As observed in these patients (*23*,*28*), because of blocking of the inflammatory response mediated by TNF-α, IL-1, or IL-6 cytokines, these patients might not have high fever and raised CRP, a fact of which clinicians should be aware (*20*–*24*,*29*). Regardless of active disease and combined immunosuppressive therapy, patients 1 and 2 (i.e., those not undergoing HSCT) had preserved inflammatory responses (Table 2) and appropriate routine immunologic testing (data not shown).

### CVC-Related Bacterial Infections

Three patients died of CVC-related bacterial sepsis with α-hemolytic *Streptococcus* and coagulase-negative *Staphylococcus*, common and fully sensitive organisms, despite timely administered appropriate antimicrobial therapy (*15*). Long-term CVCs are essential for patients requiring frequent blood tests and intravenous treatments. Unfortunately, CVC-related bloodstream infection is a well-recognized and potentially severe complication. Coagulase-negative *Staphylococcus* species are the most common pathogens causing CVC-related infections. Guidelines recommend treatment with 10–14 days of systemic antimicrobial drugs and antibiotic locks, but routine CVC removal is not recommended because most patients have a benign course and rarely develop sepsis or poor outcome (*15*). *S. aureus*, enteric gram-negative bacilli and *Candida* are less frequent but potentially more severe pathogens. Coagulase-negative *Staphylococcus* species (*S. epidermidis* in particular) were the most common (>50%) pathogens identified from 146 episodes of bacteremia in 64 children with primary immunodeficiencies undergoing HSCT in Great North Children’s Hospital, whereas *Enterococcus* species, gram-negative organisms, and *Candida* were isolated only in few cases each (*30*). Most (80%) episodes were successfully treated with appropriate systemic and antibiotic locks; CVC was removed in 12 patients, and the only death resulted from overwhelming *C. albicans* infections despite CVC removal (*30*). Contrary to that study, severe and life-threatening CVC-related sepsis caused by *S. epidermidis* has been reported in a significant percentage of children with systemic vasculitis treated with infliximab and combined immunosuppressive and/or antiinflammatory therapies (*31*). Two deaths from CVC-related sepsis resulting from coagulase-negative *Staphylococcus* and combined *Escherichia coli* and *Candida* infection were reported from a cohort of children with inflammatory bowel disease treated with adalimumab in combination with other immunosuppressive medications (*32*). This high risk for severe CVC-related infections associated with active disease that requires multiple immunomodulatory therapies was also reported from a group of children with pediatric rheumatic disease–related complications admitted to PICU, with substantially high rate of death (50%), of which 44% resulted from multiorgan failure (*33*).

### MAS versus Multiorgan Failure

Because the 3 patients we describe who died of CVC-related bacterial sepsis manifested unusually severe and rapidly progressing multiorgan failure, we considered the possibility of MAS in the differential diagnoses (*14*,*16*,*33*,*34*). MAS is a well-recognized major risk factor for death in children with JIA admitted to PICU (*33*,*34*). A recent international study of a large cohort of So-JIA patients identified the most common clinical features as fever, organomegaly, central nervous system involvement, and hemorrhage (*16*). The most useful laboratory parameters were thrombocytopenia; hyperferritinemia; increased liver transaminases, lactate dehydrogenase, triglycerides, and D-dimer levels; and decreased leukocyte count, erythrocyte sedimentation rate, and fibrinogen (*16*).

Some features that manifested near death, such as persisting fever, falling neutrophil and platelet counts, and increased serum transaminase levels (Table 2; patient 1 on March 1) suggest MAS in patient 1 (*16*). Unfortunately, not all of the laboratory parameters highlighted as essential for the clinical diagnosis of MAS were available (*14*) (Table 2). However, the fact that she collapsed after CVC was accessed and that α-hemolytic *Streptococcus* grew from the CVC line tip, lung tissue, and pleural effusion samples after death favors infection as the cause of death. In patient 2, persisting fever, hepatomegaly, and high serum ferritin level suggested MAS, but increasing platelet and neutrophil counts, erythrocyte sedimentation rate, and fibrinogen and normal liver transaminase levels did not support MAS (*14*) (Table 2; patient 2 on June 6). Although rapid deterioration to terminal multiorgan failure was associated with falling platelet count, deranged liver function, and clotting (Table 2; patient 2 on June 10), the high neutrophil count suggested overwhelming fungal infection (which cannot be ruled out because autopsy was not performed), regardless of negative fungal cultures. In patient 4, unusually severe, progressive multiorgan failure developed after CVC-related sepsis, but normal liver function results and clotting and adequate CRP response did not support MAS. Features of macrophage activation in bone marrow biopsy were seen later in the course of progressive multiorgan failure and in parallel with unusual hyperinflammation, suggesting possible fungal infection. However, multiple cultures from different sites remained negative, and autopsy confirmed severe pancreatitis. In patient 3, disseminated adenovirus and EBV infection/reactivation during HSCT led to bone marrow aplasia. In the terminal stage of multiorgan failure with *A. fumigatus* infection, results of liver function and clotting tests were normal, and inflammatory markers were raised (erythrocyte sedimentation rate 80 mm/h [Westergren method; reference 1–10 mm/h]; CRP 200 mg/L [reference 0–5 mg/L]; ferritin 11,000 μg/L [reference 20–60 μg/L]).

## Deaths and Reporting Deaths

Although the death rate for JIA has decreased since the 1970s, 1 of 2 recent studies referring to the period before the use of biological DMARDs reported a standardized mortality ratio of 3.4 (95% CI 2.0–5.5) for boys and 5.1 (95% CI 3.2–7.8) for girls (*35*). This nationwide cohort study from Scotland (1,246 children with JIA during 1981–2000) reported 39 JIA-associated deaths, for which the most common causes were the underlying disease (9 cases), circulatory complications (8 cases), and respiratory complications (6 cases) (*35*). In the United States, the standardized mortality ratio for the JIA pediatric rheumatology mortality database (9,604 children with JIA during 1992–2001) was lower at 1.8 (95% CI 0.66–3.92), with 6 of 19 reported deaths occurring among children with So-JIA caused by MAS and heart failure (2 each) and infection and secondary malignancy (1 each) (*36*).

In addition to uncontrolled disease activity and its complications, in particular amyloidosis in the past and MAS today, several recent reviews highlighted the substantial risk for death from severe infections in children with JIA who are receiving biological DMARDs (*7*,*8*). Most of these children were treated with multiple and combined classical DMARDs before or alongside biological DMARDs. Although the range of causing pathogens is broad, pyogenic bacteria and herpes viruses were the most common (*7*,*8*). An unexpectedly high death rate (68% [17/25]) in a group of children with refractory So-JIA associated with pulmonary complications was recently reported from an international group of 25 patients (*19*). Although disease onset ranged from the 1980s onward and most children in the cohort had received multiple immunosuppressive and antiinflammatory drugs, 19 (76%) diagnoses were made after 2000; contrary to previous reports (*35*,*36*), 68% had been exposed to biological DMARDs (*19*). Strikingly, the calculated death rate for these 19 patients increased by almost 50% over the figure recently reported from the US pediatric rheumatology mortality database for the period up to 2000, before the use of biological DMARDs (*36*).

The regional pediatric rheumatology service at Great North Children’s Hospital is closely linked to the national center providing expertise in investigating and treating children with primary immunodeficiencies for northern England and a leading center for HSCT in children with severe rheumatic diseases. This database holds ≈1,250 children in whom JIA has been diagnosed and who were treated and followed during 1994–2013; each year, 50–80 new patients are referred. The calculated death rate for children in this cohort is 0.032% (4 patients died during this period). Three had So-JIA and 1 rheumatoid factor + poly-JIA, all with progressive polyarthritis and poorly controlled systemic disease for many years, including fever, rash, organomegaly, and high acute-phase reactants, with features of MAS in 2 So-JIA patients (*14*,*16*). All died during the early 2000s after long-term treatment with multiple and combined conventional and biological DMARDs: corticosteroids and methotrexate (all 4 children), cyclosporine and leflunomide (1 each); anti–TNF-α agents (3 etanercept, 4 infliximab); IL-1 blocking agent (2 anakinra); and B-cell–depleting (anti-CD20) monoclonal antibody (1 rituximab). Although considered in all 4, only 2 patients underwent autologous T-cell–depleted HSCT (*10*,*11*). With the expertise in pediatric rheumatology, immunology, and infectious diseases available in centers best placed to care for these most severe and often refractory cases, fatalities are rare but do occur.

Monitoring the safety and reporting the side effects, including severe infections and deaths, of new biological DMARDs is a priority of national and international patient registries and multicenter, international collaborative research consortiums, such as the Childhood Arthritis and Rheumatology Research Alliance, Pediatric Rheumatology International Trials Organisation, Pediatric Rheumatology Collaborative Study Group, and Single Hub and Access Point of Care for Pediatric Rheumatic Diseases in Europe (*37*,*38*). However, reporting of deaths is still inconsistent. In children with JIA treated with multiple DMARDs alongside anti–TNF-α biologicals, a systematic review from 2013 reported 4 deaths, 3 of which were associated with severe infections: 2 treated with etanercept (group A *Streptococcus*–related purpura fulminans) and 1 with adalimumab (bacterial sepsis); for 1 treated with infliximab, infection cause was not given (*27*). However, Hashkes et al. (*8*) commented on 6 deaths, all in children treated with combined DMARDs, including anti–TNF-α agents, and associated with serious infections: 3 treated with etanercept (1 each with suspected sepsis, MAS, and tuberculosis [previously treated with infliximab]); 2 with infliximab (sepsis); and 1 with adalimumab (MAS and interstitial pneumonia). Furthermore, neither Woerner and Ritz (*7*) nor Swart et al. (*24*) referred to severe infections and infection-related deaths associated with biological DMARDs from 2 clinical trials reported in 2012 (*39*,*40*). Six deaths were reported in trials of the anti–IL-6 agent tocilizumab, including 1 each from probable streptococcal sepsis and MAS (other listed causes were traffic accident, pulmonary hypertension [2 cases], and pneumothorax) (*40*). Two deaths were reported in a trial of long-acting anti–IL-1 agent canakinumab, both from MAS (1 was previously treated with anakinra and tocilizumab) (*39*). All these patients had active disease and had been treated with a combination of multiple DMARDs before or at death; some were reported as part of the So-JIA cohort associated with pulmonary complications and high death rate (*19*). The most recent report from the United States for 2008–2012 highlighted 7 deaths (1 from an accident) in children with JIA treated with multiple DMARDs (methotrexate and steroids), including biologicals (4 anakinra, 1 each etanercept and infliximab), of which 3 were associated with severe infections (multiorgan failure from disseminated tuberculosis, viral illness, and sepsis) (*38*).

## Conclusions

Three patients with refractory JIA reported here died with evidence of CVC-related bacterial sepsis caused by common pathogens (α-hemolytic *Streptococcus* and coagulase-negative *Staphylococcus*): 2 while receiving high-dose systemic corticosteroids and methotrexate and after recent exposure to the anti–TNF-α biological DMARD infliximab; 1 during HSCT procedure. As has been previously reported, we observed unusual severity of septic shock and rapid progression to multiorgan failure despite timely and appropriate antimicrobial treatment (*31*–*33*). Patient 4 died of disseminated adenovirus and EBV infection during HSCT procedure.

Evidence from clinical trials facilitated by multicenter international collaborative research enabled introduction of biological DMARDs in the treatment of rheumatic diseases in children and unprecedented improvement in their care during the past 20 years (*1*–*4*,*41*,*42*). However, severe infections are emerging as an important risk factor for death among children with JIA treated with combined and multiple conventional and new biological DMARDs (*8*,*19*,*37*,*38*). Accurately reporting all cases of severe infections and especially deaths in these children is of paramount importance, as was highlighted a decade ago (*25*). Although monitoring safety and reporting side effects of new biological DMARDs is in place and improving, we note marked inconsistency in the current literature (*7*,*8*,*19*,*24*,*27*,*38*–*40*).

## References

[1] Prakken B, Albani S, Martini A. Juvenile idiopathic arthritis. Lancet. 2011;377:2138–49. 10.1016/S0140-6736(11)60244-4 21684384

[2] Hinze C, Gohar F, Foell D. Management of juvenile idiopathic arthritis: hitting the target. Nat Rev Rheumatol. 2015;11:290–300. 10.1038/nrrheum.2014.212 25561365

[3] Ringold S, Weiss PF, Beukelman T, Dewitt EM, Ilowite NT, Kimura Y, ; American College of Rheumatology. 2013 update of the 2011 American College of Rheumatology recommendations for the treatment of juvenile idiopathic arthritis: recommendations for the medical therapy of children with systemic juvenile idiopathic arthritis and tuberculosis screening among children receiving biologic medications. Arthritis Care Res (Hoboken). 2013;65:1551–63. 10.1002/acr.22087 24078300 PMC5408573

[4] Southwood TR. Paediatric rheumatic disease: Treatment of JIA in the biologic era: what are we waiting for? Nat Rev Rheumatol. 2014;10:132–4. 10.1038/nrrheum.2014.14 24514914

[5] Otten MH, Prince FH, Anink J, Ten Cate R, Hoppenreijs EP, Armbrust W, Effectiveness and safety of a second and third biological agent after failing etanercept in juvenile idiopathic arthritis: results from the Dutch National ABC Register. Ann Rheum Dis. 2013;72:721–7. 10.1136/annrheumdis-2011-201060 22730374

[6] Record JL, Beukelman T, Cron RQ. Combination therapy of abatacept and anakinra in children with refractory systemic juvenile idiopathic arthritis: a retrospective case series. J Rheumatol. 2011;38:180–1. 10.3899/jrheum.100726 21196588

[7] Woerner A, Ritz N. Infections in children treated with biological agents. Pediatr Infect Dis J. 2013;32:284–8. 10.1097/INF.0b013e3182833cbb 23558323

[8] Hashkes PJ, Uziel Y, Laxer RM. The safety profile of biologic therapies for juvenile idiopathic arthritis. Nat Rev Rheumatol. 2010;6:561–71. 10.1038/nrrheum.2010.142 20808294

[9] Abinun M. Haematopoietic stem cell transplantation for rheumatological conditions. Paediatr Child Health. 2011;21:558–62 . 10.1016/j.paed.2011.06.003

[10] Abinun M, Flood TJ, Cant AJ, Veys P, Gennery AR, Foster HE, Autologous T cell depleted haematopoietic stem cell transplantation in children with severe juvenile idiopathic arthritis in the UK (2000-2007). Mol Immunol. 2009;47:46–51. 10.1016/j.molimm.2008.12.029 19203795

[11] Milanetti F, Abinun M, Voltarelli JC, Burt RK. Autologous hematopoietic stem cell transplantation for childhood autoimmune disease. Pediatr Clin North Am. 2010;57:239–71. 10.1016/j.pcl.2009.12.003 20307720

[12] Myers A, Clark J, Foster H. Tuberculosis and treatment with infliximab. N Engl J Med. 2002;346:623–6. 10.1056/NEJM200202213460815 11859881

[13] Consolaro A, Ruperto N, Bracciolini G, Frisina A, Gallo MC, Pistorio A, ; Paediatric Rheumatology International Trials Organization (PRINTO). Defining criteria for high disease activity in juvenile idiopathic arthritis based on the juvenile arthritis disease activity score. Ann Rheum Dis. 2014;73:1380–3. 10.1136/annrheumdis-2013-204186 24347571

[14] Ravelli A, Minoia F, Davì S, Horne A, Bovis F, Pistorio A, 2016 classification criteria for MAS complicating SO-JIA: EULAR/ACR/PPRINTO Initiative. Arthritis Rheumatol. 2016;68:566–76. 10.1002/art.39332 26314788

[15] Mermel LA, Allon M, Bouza E, Craven DE, Flynn P, O’Grady NP, Clinical practice guidelines for the diagnosis and management of intravascular catheter-related infection: 2009 Update by the Infectious Diseases Society of America.[ [Erratum in: Clin Infect Dis. 2010;50:457. Erratum in: Clin Infect Dis. 2010;50:1079. ]. Clin Infect Dis. 2009;49:1–45. 10.1086/599376 19489710 PMC4039170

[16] Minoia F, Davì S, Horne A, Demirkaya E, Bovis F, Li C, ; Pediatric Rheumatology International Trials Organization; Childhood Arthritis and Rheumatology Research Alliance; Pediatric Rheumatology Collaborative Study Group; Histiocyte Society. Clinical features, treatment, and outcome of macrophage activation syndrome complicating systemic juvenile idiopathic arthritis: a multinational, multicenter study of 362 patients. Arthritis Rheumatol. 2014;66:3160–9. 10.1002/art.38802 25077692

[17] Lovell DJ, Zaoutis TE, Sullivan K. Immunosuppressants, infection, and inflammation. Clin Immunol. 2004;113:137–9. 10.1016/j.clim.2004.03.010 15451468

[18] Beukelman T, Xie F, Chen L, Baddley JW, Delzell E, Grijalva CG, ; SABER Collaboration. Rates of hospitalized bacterial infection associated with juvenile idiopathic arthritis and its treatment. Arthritis Rheum. 2012;64:2773–80. 10.1002/art.34458 22569881 PMC3409300

[19] Kimura Y, Weiss JE, Haroldson KL, Lee T, Punaro M, Oliveira S, ; Childhood Arthritis Rheumatology Research Alliance Carra Net Investigators. Pulmonary hypertension and other potentially fatal pulmonary complications in systemic juvenile idiopathic arthritis. Arthritis Care Res (Hoboken). 2013;65:745–52. 10.1002/acr.21889 23139240 PMC4476507

[20] van de Vosse E, van Dissel JT, Ottenhoff TH. Genetic deficiencies of innate immune signalling in human infectious disease. Lancet Infect Dis. 2009;9:688–98. 10.1016/S1473-3099(09)70255-5 19850227

[21] Abinun M. An overview of infectious complications in children on new biologic response-modifying agents. Ped Health. 2010;4:509–17 . 10.2217/phe.10.57

[22] Maródi L, Casanova JL. Primary immunodeficiencies may reveal potential infectious diseases associated with immune-targeting mAb treatments. J Allergy Clin Immunol. 2010;126:910–7. 10.1016/j.jaci.2010.08.009 20943260

[23] Puel A, Picard C, Lorrot M, Pons C, Chrabieh M, Lorenzo L, Recurrent staphylococcal cellulitis and subcutaneous abscesses in a child with autoantibodies against IL-6. J Immunol. 2008;180:647–54. 10.4049/jimmunol.180.1.647 18097067

[24] Swart JF, de Roock S, Wulffraat NM. What are the immunological consequences of long-term use of biological therapies for juvenile idiopathic arthritis? Arthritis Res Ther. 2013;15:213. 10.1186/ar4213 23731900 PMC4060240

[25] Fitch PG, Cron RQ. Septic abscess in a child with juvenile idiopathic arthritis receiving anti-tumor necrosis factor-alpha. J Rheumatol. 2006;33:825, author reply 826–7. 16583487

[26] Renaud C, Ovetchkine P, Bortolozzi P, Saint-Cyr C, Tapiero B. Fatal group A *Streptococcus* purpura fulminans in a child receiving TNF-α blocker. Eur J Pediatr. 2011;170:657–60. 10.1007/s00431-010-1341-1 21063727

[27] Toussi SS, Pan N, Walters HM, Walsh TJ. Infections in children and adolescents with juvenile idiopathic arthritis and inflammatory bowel disease treated with tumor necrosis factor-α inhibitors: systematic review of the literature. Clin Infect Dis. 2013;57:1318–30. 10.1093/cid/cit489 23899685 PMC3888230

[28] Elwood RL, Pelszynski MM, Corman LI. Multifocal septic arthritis and osteomyelitis caused by group A *Streptococcus* in a patient receiving immunomodulating therapy with etanercept. Pediatr Infect Dis J. 2003;22:286–8. 10.1097/01.inf.0000055092.90609.e5 12664882

[29] Henderson C, Goldbach-Mansky R. Monogenic IL-1 mediated autoinflammatory and immunodeficiency syndromes: finding the right balance in response to danger signals. Clin Immunol. 2010;135:210–22. 10.1016/j.clim.2010.02.013 20353899 PMC2856775

[30] Cole TS, Rogerson E, Collins J, Galloway A, Clark J. Central venous catheter-related blood stream infections in children undergoing hematopoietic stem cell transplant for primary immunodeficiency and other nonmalignant disorders. Pediatr Infect Dis J. 2011;30:1098–100. 10.1097/INF.0b013e31822940a8 21730885

[31] Eleftheriou D, Melo M, Marks SD, Tullus K, Sills J, Cleary G, Biologic therapy in primary systemic vasculitis of the young. Rheumatology (Oxford). 2009;48:978–86. 10.1093/rheumatology/kep148 19535611

[32] Russell RK, Wilson ML, Loganathan S, Bourke B, Kiparissi F, Mahdi G, A British Society of Paediatric Gastroenterology, Hepatology and Nutrition survey of the effectiveness and safety of adalimumab in children with inflammatory bowel disease. Aliment Pharmacol Ther. 2011;33:946–53. 10.1111/j.1365-2036.2011.04603.x 21342211

[33] Radhakrishna SM, Reiff AO, Marzan KA, Azen C, Khemani RG, Rubin S, Pediatric rheumatic disease in the intensive care unit: lessons learned from 15 years of experience in a tertiary care pediatric hospital. Pediatr Crit Care Med. 2012;13:e181–6. 10.1097/PCC.0b013e318238955c 22561277

[34] Shulman AI, Punaro M. Critical care of the pediatric patient with rheumatic disease. Curr Opin Pediatr. 2011;23:263–8. 10.1097/MOP.0b013e328346198f 21467937

[35] Thomas E, Symmons DP, Brewster DH, Black RJ, Macfarlane GJ. National study of cause-specific mortality in rheumatoid arthritis, juvenile chronic arthritis, and other rheumatic conditions: a 20 year followup study. J Rheumatol. 2003;30:958–65. 12734889

[36] Hashkes PJ, Wright BM, Lauer MS, Worley SE, Tang AS, Roettcher PA, Mortality outcomes in pediatric rheumatology in the US. Arthritis Rheum. 2010;62:599–608. 20112378 10.1002/art.27218

[37] Lionetti G, Kimura Y, Schanberg LE, Beukelman T, Wallace CA, Ilowite NT, Using registries to identify adverse events in rheumatic diseases. Pediatrics. 2013;132:e1384–94. 10.1542/peds.2013-0755 24144710 PMC3813393

[38] Ringold S, Hendrickson A, Abramson L, Beukelman T, Blier PR, Bohnsack J, Novel method to collect medication adverse events in juvenile arthritis: results from the childhood arthritis and rheumatology research alliance enhanced drug safety surveillance project. Arthritis Care Res (Hoboken). 2015;67:529–37. 10.1002/acr.22487 25331530

[39] Ruperto N, Brunner HI, Quartier P, Constantin T, Wulffraat N, Horneff G, ; PRINTO; PRCSG. Two randomized trials of canakinumab in systemic juvenile idiopathic arthritis. N Engl J Med. 2012;367:2396–406. 10.1056/NEJMoa1205099 23252526

[40] De Benedetti F, Brunner HI, Ruperto N, Kenwright A, Wright S, Calvo I, ; PRINTO; PRCSG. Randomized trial of tocilizumab in systemic juvenile idiopathic arthritis. N Engl J Med. 2012;367:2385–95. 10.1056/NEJMoa1112802 23252525

[41] van Royen-Kerkhof A, Vastert BS, Swart JF, Wulffraat NM. Biologic treatment of pediatric rheumatic diseases: are we spoilt for choice? Immunotherapy. 2014;6:1–3. 10.2217/imt.13.153 24341875

[42] Wahezi DM, Ilowite NT. Juvenile idiopathic arthritis: an update on current pharmacotherapy and future perspectives. Expert Opin Pharmacother. 2013;14:975–89. 10.1517/14656566.2013.783569 23528005

